# Irregularity of the Teeth

**Published:** 1848-04

**Authors:** J. Taft

**Affiliations:** Surgeon Dentist, Xenia, Ohio


### ARTICLE III.

*Irregularity of the Teeth.* By J. TAFT, Surgeon Dentist, Xenia, Ohio

THE proper treatment of irregularity of the teeth is not a matter of minor importance either to the dentist, or such as are afflicted with it. The following case came under my care, during the winter of 1843, Mr. W. applied to me for advice in regard to the irregularity of his son's teeth, who was about nine year's old. Upon examination, I found the following arrangement of his teeth to exist. The medial side of the left central incisor was turned anteriorly, so that its cutting edge formed an angle of fifty degrees from the proper circle or line of the cutting edges of the other incisores, its lateral edge inward; the central edge of the right incisor was directly against the centre of the posterior face of the left incisor. The right central incisor pointed about fifteen degrees to the interior of the true dental circle. The cutting edge of the left lateral incisor was turned so as to form an angle of eighty degrees from its true position in the circle; its proper anterior side, or edge, was turned inward, and the posterior side, or edge, was turned outward, standing with the anterior surface against the lateral edge of the left central incisor. The right lateral incisor, with its cutting edge, at an angle of forty degrees, its posterior edge, or side, pointing outward, and its anterior inward. The posterior primary teeth were all remaining, some quite loose. There may not be any thing in the treatment of this case, new or interesting to the profession, but I will give it. I took a cast of the part, and formed a heavy narrow plate, which was clasped to the molar teeth, and then soldered flat wire upon the plate at the proper points and turned them into hooks which caught upon the external edges of the irregular teeth, and every two days the hooks were shortened, until the teeth were all brought against the external edge of the plate, which was the exact form of the proper dental arch. The right central incisor was easily moved out by the pressure of the plate. While this was going on I used a strong stimulant to induce energetic action of the absorbing vessels. By this treatment the desired end was accomplished in six weeks.

